# Comparison of various methodological approaches to model asbestos thresholds for mesothelioma

**DOI:** 10.3389/fpubh.2025.1569343

**Published:** 2025-05-13

**Authors:** Julie E. Goodman, Andrey Korchevskiy, Ann G. Wylie

**Affiliations:** ^1^Gradient, Boston, MA, United States; ^2^Chemistry & Industrial Hygiene, Inc., Lakewood, CO, United States; ^3^Department of Geology, University of Maryland, College Park, MD, United States

**Keywords:** threshold, mesothelioma, asbestos, chrysotile, amphibole, dose-response

## Abstract

**Background:**

There is evidence to support several modes of action (MoAs), and particularly non-genotoxic MoAs, for mesothelioma induced by asbestiform elongate mineral particles (EMPs). In turn, these MoAs provide biological support for dose-response relationships that are non-linear and that include a threshold. However, statistical models of human data have not adequately addressed threshold dose-response relationships for asbestiform EMPs and mesothelioma. In addition, unlike other carcinogens, asbestiform EMPs are not uniform materials and display a range of properties.

**Objectives:**

Our objective was to review various approaches for applying threshold dose-response models to asbestiform EMPs and mesothelioma.

**Materials and methods:**

We collected data from several sources, including the Surveillance, Epidemiology, and End Results (SEER) Program and published case-control studies, cohort studies, and a meta-analysis that evaluated various mineral types of asbestos and mesothelioma risk. Several threshold-based models were fit to the available data. We also evaluated thresholds for certain fiber characteristics.

**Results:**

Certain characteristics of asbestiform EMPs, such as width, length, and surface area, likely have thresholds for mesothelioma. Theoretical models and models based on epidemiology data supported thresholds. A Monte Carlo evaluation of the threshold hypothesis for mesothelioma in a meta-analysis of occupational exposures to various mineral fiber types, using a cumulative exposure metric, demonstrated the likelihood of a threshold to be 72% for non-textile chrysotile, 80.9% for textile chrysotile, 84% for amosite, and 60% for crocidolite. A multi-stage clonal expansion (MSCE) model applied to the SEER mesothelioma registry data demonstrated a good fit with the inclusion of a threshold by a surrogate predictor of cumulative exposure to amphiboles. Finally, lung burden studies also support a threshold. Our preliminary estimate of a central-tendency cumulative exposure threshold level for non-textile chrysotile is ~90 f/cc-years. Based on our proposed approach, we suggest thresholds of 1.04 f/cc-years for amosite, 0.25 f/cc-years for crocidolite, and 4.3–10.9 f/cc-years for tremolite. Future studies should be conducted to support these estimates.

**Conclusions:**

While uncertainties remain, many angles of scientific evidence support the existence of mineral-specific thresholds for mesothelioma.

## Introduction

Malignant mesothelioma is a rare disease. While some cases are spontaneous or due to other causes (*e.g*., radiation), most are associated with asbestos exposure (1, 2). However, in asbestos-exposed populations, only a small fraction of heavily exposed individuals develop mesothelioma (3), and virtually all populations in rural and urban areas of developed countries have been exposed to at least some level of background asbestos (4, 5). Asbestos fibers are also routinely present in the lungs of people in the general population (4, 5). There has been discussion about the ability of one fiber of asbestos to cause mesothelioma, but the arguments in support of this hypothesis are nonsensical, in particular because such an exposure would not be measurable, comprising about one-billionth of the background exposure for millions people who never develop mesothelioma during their lifetimes.

In their famous study, Hodgson and Darnton (6) cited a United Kingdom Health and Safety Executive review that suggested that there is a toxicological basis for a threshold for asbestos-induced lung cancer (7). The biological basis of a threshold for asbestos exposure in mesothelioma is rooted in the understanding of the mechanisms of the development for this type of tumor. One of the suggested mechanisms is oxidative stress, which can result directly from endogenous iron in asbestos fibers or indirectly from inflammation following direct activation of inflammatory cells or from frustrated phagocytosis (*i.e*., inefficient clearing of long fibers by macrophages) (8–14). Bioavailable iron on the surface of the fiber can promote the conversion of hydrogen peroxide to hydroxyl radicals, other reactive oxygen species, or reactive nitrogen species (9, 12, 13, 15, 16). This effect is dose-dependent; asbestos fibers with higher iron contents produce more reactive oxygen species than low-iron fibers (12, 13, 17). Oxidative stress due to iron is primarily associated with amphibole asbestos fibers because of their much higher iron content (27.3%) compared to chrysotile (0.7-2%) (8, 9, 12, 18, 19). Also, in many toxicological models for asbestos-related mesothelioma, chronic inflammation plays a pivotal role (20). Many toxicological models for asbestos-induced mesothelioma imply a threshold.

Hodgson and Darnton (6) argued that there is no evidence for a threshold for mesothelioma risk. They also emphasized that a threshold cannot be determined based on minimum exposure levels determined in mesothelioma cases. However, a non-threshold hypothesis also cannot be confirmed based on limited epidemiology information. Notably, average cumulative exposure levels with elevated mesothelioma mortality in the most recent study by Darnton (21) exceeded 16.4 fibers per cubic centimeter-years (f/cc-years) for crocidolite, 23.6 f/cc-years for amosite, 28 f/cc-years for textile chrysotile, and 46 f/cc-years for non-textile chrysotile.

Even if only biological studies can further specifically address threshold exposure levels and fiber characteristics necessary for mesothelioma to develop, one can analyze quantitative principles of thresholds based on available data. That is, characteristics of dose-response curves at higher doses can be used to make possible judgements of their behavior at lower doses, as demonstrated in benchmark dose theory (22).

In this paper, we develop and compare several approaches for testing the threshold hypothesis for asbestos and mesothelioma. We demonstrate that existing epidemiology data do not contradict the threshold hypothesis. We used chrysotile epidemiology data in several tested models, and then extrapolated this to other mineral fibers based on potency. Future studies that could address thresholds for mesothelioma are also discussed.

## Methods

The data for malignant mesothelioma mortality in several mining and general industry chrysotile cohorts were used for testing dose-response models. We did not use textile cohorts because Darnton (21) demonstrated that mesothelioma potency for chrysotile in asbestos textile cohorts was statistically significantly higher than that for mining and general industry cohorts. Various reasons exist for this difference; one is significant uncertainty with amphibole exposure in the textile cohorts (23). Thresholds calculated here should not be applied to the textile cohorts without correction factors, but the methodology proposed can be tested on other cohorts/mineral types.

A total of six cohorts were included in our analysis of mining and general industry chrysotile, five of which were utilized by Darnton (21) in her recent publication. The sixth cohort, which included miners and millers in Russia, was evaluated by the International Agency for Research on Cancer (IARC) and other research organizations and published by Schüz et al. (24). These cohorts are characterized in Table 1.

**Table 1:** Epidemiology data used for the analysis.

**Cohort**	**Avg. exposure duration (years)**	**Avg. exposure intensity (PCM f/cc)**	**Cumulative exposure (PCM f/cc-years)**	**Avg. age at exposure onset (years)**	**Mesothelioma cases**	**Total expected mortality^a^**	**Excess mesothelioma mortality**	**Survival rate function (S)^b^**
Québec miners and millers	35	17.14	600	23	33	5,913	0.0054	0.995
Balangero miners and millers^c^	16.95	42.5	721	27	7	549	0.0128	0.987
Quinghai miners	27.3	4.39	120	21.7	0	366	0	1
New Orleans asbestos cement workers	3.9	5.64	22	27	0	500	0	1
Connecticut asbestos friction products workers	8.04	5.72	46	31	2	274	0.00072	0.999
Russian miners and millers^d^	15	2.24	33.6	24	13	10,351	0.0012	0.998

^a^Adjusted to onset of exposure age of 30 years.

^b^Fraction of the population without mesothelioma.

^c^Updated information on chrysotile exposures in Balangero workers was based on the updated value from Korchevskiy and Wylie (31).

^d^The total expected mortality of workers in the Russian cohort was not found in the published data. It was approximated based on available information regarding the cohort size.

Avg., Average; f/cc, Fibers Per Cubic Centimeter; PCM, Phase-Contrast Microscopy.

The average exposure duration and age of mesothelioma diagnosis for various cohorts were derived from original published sources, as was previously demonstrated by Korchevskiy and Korchevskiy (25). These data, along with the number of mesothelioma cases and total expected mortality (characteristic of cohort size and age distribution) are shown in Table 1.

To model mesothelioma risk in the United States (US), data from the Surveillance, Epidemiology, and End Results (SEER) cancer registry for the period 1973–2018 were used. For asbestos consumption, we utilized data from the United States Geological Survey (26). We used Statistica 14.0 for statistical modeling, Wolfram Alpha for calculus, and Crystal Ball for Excel for statistical simulation.

## Results

### Mineral fiber characteristic thresholds

Although thresholds for asbestos and mesothelioma are often considered in the context of exposure intensity (*e.g*., mean or cumulative f/cc or f/cc-years), they can also be considered in the context of mineral fiber characteristics, as not all fiber types or fiber sizes are likely to produce mesothelioma in humans. Exposure to non-mesotheliomagenic fibers by definition is not likely to cause this type of cancer, at any exposure, while other fiber types and sizes are only likely to cause mesothelioma at very high levels of exposure. Therefore, there are properties of mesotheliomagenic fibers that have thresholds. For example, one threshold often accepted is a length threshold of 5 μm. Some have also proposed a threshold length of 20 μm (27). Other properties include fibril width, rigidity, solubility, and interfibrillar bonding. Notably, there is a significant difference in mesothelioma potency for various types and sources of fibers, and elongate cleavage fragments are counted as fibers but have no known potency for mesothelioma. Furthermore, what is termed “asbestos” exhibits a wide range in properties, each of which may include a threshold for carcinogenic potency. In this way, asbestos is unlike any other carcinogen, and the approach to understanding the risks it presents are different.

#### Size

Fiber dimensions (length and width) impact health risks because they affect respirability, deposition, and biopersistence in the lung (28). They also affect a fiber's ability to translocate to other tissue, such as the pleura.

Three mineral types of asbestos—chrysotile, amosite, and crocidolite—commercially produced from different mines, were used extensively as building and insulation materials in the US; chrysotile is a serpentine asbestos, and amosite and crocidolite are amphibole asbestos. Chrysotile was by far the most common type of fiber used in the US, and most people still have periodic exposures to chrysotile fibers in the urban environment. These three asbestos sources have different ranges in fibril size. Because they also have different potencies for mesothelioma that correlate with fibril size, there are likely thresholds for fiber size for which potency is negligible.

The average width of chrysotile fibers in lung tissue is commonly reported as 60 nm; the average fibril width of Canadian chrysotile from the Thetford mine area is 25 nm (29). A fiber with the average width of 60 nm is likely composed of several fibrils. Amphibole fibrils generally exhibit higher variability in width than chrysotile fibrils. From lung burden data, and studies that have looked directly at cross sections of asbestos, we know that the most uniform amphibole is crocidolite with average fibrils width widths <100 nm; for amosite, average fibril widths could be as large as about 200 nm or more, and anthophyllite fibrils can have widths of 400 nm or more. All these commercial forms of amphibole asbestos have a proportion of fibrils longer than 5 μm that are <150 nm in width, but the proportion varies (30, 31). As fibril sizes change, properties change. For example, the luster of amphibole fibers is no longer silky when fibril widths exceed a wavelength of light and amphibole fibrils 1–2 um in width have lost all excess tensile strength, are glassy, and are brittle. It seems likely that between the smallest, rigid fibrils (about 60 nm) and 1,000 nm there is a maximum width threshold above which fibers are non-mesotheliomagenic and below which they are. According to the Occupational Safety and Health Administration (OSHA) (32), fibers >3 μm in width do not pose human health risks, but the maximum width is probably smaller than 3,000 nm.

Length is also a variable that likely has a threshold for mesothelioma. We find that many studies assume a threshold for length that is 5 μm because only fibers longer than 5 μm are counted in occupational exposure assessments. Barlow et al. (27) concluded that “there is very little, if any, risk associated with exposure to fibers shorter than 5 μm.” Similarly, OSHA (32) concluded that evidence indicates that exposure to fibers with lengths >5 μm increases risk for asbestosis, mesothelioma, and lung cancer vs. exposure to fibers <2.5 μm long [see also Wylie and Korchevskiy (30)]. Fibers a few μm in length are similar to other common mineral dusts in size and are readily phagocytized by macrophages and cleared (33).

On the other hand, long, respirable fibers frustrate macrophage removal, and they can translocate to lower airways, where the longer they are they less likely they are to be cleared (28, 33–36).

It seems clear that very short fibers are below the length threshold for mesothelioma. Chrysotile asbestos fibers recovered from lung tissue are reported in many studies to have an average geometric mean (GM) length of about 1 μm, so many chrysotile fibrils will likely fall below the length threshold. Amphibole recovered from lung tissue has average GM lengths that range from about 2 to 5 μm, and many most likely will fall above a length threshold. If fibers must be in direct contact with mesothelial cells to induce mesothelioma, then there will be an upper limit of length as well. For lung burden, the longest fibers reported are about 100 μm. Fibers of this length will not likely translocate to the pleura, Therefore, the threshold for length likely falls between about 5 μm and 100 μm.

#### Mineral solubility

It is generally well recognized that mineral fibers that cause mesothelioma must be retained to interact with the target tissue. Minerals that are soluble in the chemical environment of the lung will not be retained and will not translocate to the pleura. Silicate minerals are highly variable and range in solubility and their expected half-lives following inhalation ranges from days to far longer than a lifetime (i.e., thousands of years). Surely, there is a solubility threshold below which potency for mesothelioma would be negligible.

Many have proposed that the solubility of chrysotile exceeds the threshold and is too high for it to be mesotheliomagenic. Another mineral that has been shown to be below the solubility threshold is wollastonite (37).

#### Rigidity

The rigidity of fibers is also a biologically important variable that ranges over an order of magnitude and affects the carcinogenic potential of durable mineral fibers. Work on carbon nanotubes (CNTs) has already demonstrated a rigidity threshold that limits their mesotheliomagenic potential. If they are less than about 35 nm in width, CNTs do not cause in mesothelioma in rats. It is likely that chrysotile fibers narrower than 60 nm, depending on length, could lack biological rigidity (38). However, more than about ~90% of amphibole fibers are wider than 60 nm and would be expected to be biologically rigid. Because rigidity is also affected by the cross-sectional shape, the relationship between width and thickness, and the value of Young's modulus,^1^ some sheet silicates fibers other they chrysotile may not meet a rigidity threshold (38).

Recently, a rigidity index was proposed to characterize the biological impact of EMPs. The threshold of 0.05 μm^2^ × GPa × 10^4^ was demonstrated for mesotheliomagenic CNTs and EMPs (39). The index is in close agreement with the analysis of biological rigidity by Fortini et al. (40), using flexural rigidity. Fortini et al. suggested classifying particles presenting a flexural rigidity lower than 10^−19^ N × m^2^ as flexible and harmless, fibers with rigidity in the order of 10^−19^ N × m^2^ as either flexible or rigid, and fibers with rigidity above 10^−19^ N × m^2^ as rigid; rigid fibers pose a hazard to macrophage.

#### Clearance rates

Clearance rates are related to the above three properties, as well as others such as surface charge, but can be considered as a measurable, mineral-specific variable that may have a threshold. How fast must fiber be cleared to eliminate mesothelioma risk?

Compared to amphibole fibers, the chemical instability and greater tendency to fragment into small pieces allow chrysotile fibers to be readily cleared from the lungs (28, 41). Studies that have evaluated inhaled chrysotile fibers in animals have reported clearance half-lives of days to weeks [e.g., (35, 42, 43)], while studies of amphiboles have reported half-lives on the order of years (42, 44–48). Boutin et al. (49) assessed fiber type in the parietal pleura, lung, and pleural black spots of humans exposed to asbestos and found that amphiboles outnumbered chrysotile in all samples. This finding is consistent with faster clearance of chrysotile than amphibole fibers. Thus, it can be concluded that chrysotile is significantly less potent than amphiboles, such that exposure to chrysotile at lower concentration rates does not significantly increase mesothelioma risk (50).

#### Combining width and length thresholds

Several researchers have reported that mesothelioma potency was most strongly associated with fibers longer than 5 μm when the width was not >0.15 μm, which has been defined as the “EMPA” category (30, 51, 52). Thus, it can be suggested that the dimensional threshold boundaries of mesothelioma potency for EMPs include EMPA fibers.

It has been demonstrated that a Dimensional Coefficient of Carcinogenicity (DCC) could be proposed for mesothelioma induction by CNTs and EMPs. DCC is a probabilistic expression that is directly proportional to surface area of particles and inversely proportional to the third power of their diameter. There is a statistically supported threshold of mesotheliomagenic fibers that are expected to have DCC ≥ 0.05, with the mixed category comprising fibers with DCC ≥ 0.01 (53).

### Theoretical model of mesothelioma threshold

Mesothelial carcinogenesis from fiber inhalation can be considered a result of several counteracting processes. Chronic inflammation is a general process that has been suggested as a core mechanism of asbestos carcinogenicity (54), but inflammation and the resulting cytotoxicity caused by asbestos exposure is expected to increase cell mortality, eradicating pre-cancerous and cancerous cells (55, 56). Thus, mesothelioma carcinogenesis may originate from immunosuppression activities or the secretion of specific “immortalization” proteins that prevent the controlled death of abnormal cells (57).

This is demonstrated in Equation 1. X is a metric of the exposure to mesotheliomagenic EMPs. At lower levels of X, EMPs do not induce carcinogenicity because inflammation, which is a key event in their MoA, is a threshold process (58, 59). At higher exposure levels, inflammation occurs, but the immune system and induced cytotoxicity does not only control the extent of inflammation, but also eliminates cells with possible pre-cancerous or cancerous changes. Only upon reaching the inflection point of the dose-response curve would X, the level of exposure, be associated with a sufficient level of immunosuppression or cell immortalization that would allow the tumor to develop and proliferate (56). We modeled these counteracting processes with the following equation:


(1) $$\begin{array}{l} P\;=\;\left(\lambda X-1\right)\;\left(1-\beta X\right)\;\alpha X \end{array}$$

where P is the probability of mesothelioma, λX-1 is the probability of inflammation, 1-βX is the probability of cell survival, and αX is the probability of secretion of immunosuppression and/or cell immortalization proteins.

**Figure 1:**
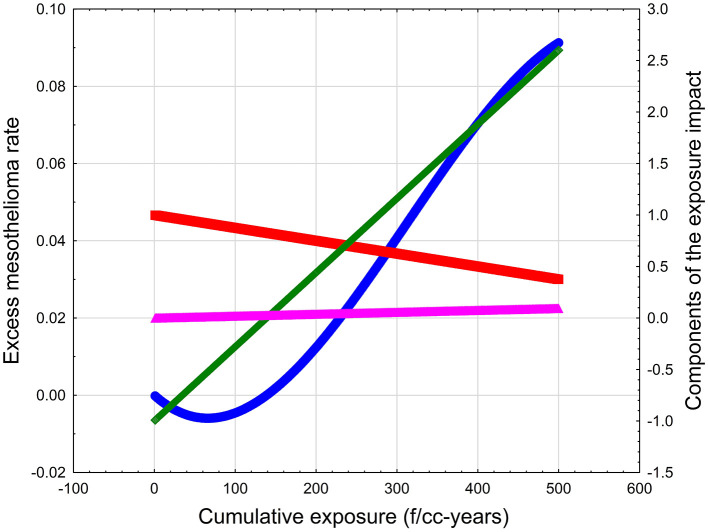
The illustration of the conceptual model of mesothelioma threshold. Blue line—Excess mesothelioma rate. Green line—Probability of inflammation. Red line—Probability of cell survival. Magenta line—Probability of immunosuppression (cell immortalization).

Equation 1 is conceptual and is not expected to be fully demonstrated using epidemiology data. However, we can use various parameters to demonstrate the behavior of P as a function of X. For a hypothetical type of EMP, we can assume that X is a cumulative exposure, or a product of exposure intensity in f/cc and the duration of exposure in years. It is difficult to estimate specific values for λ, β, and α, but some arbitrary combinations of the coefficients in Equation 1 can be demonstrated to produce threshold effect. For example, for λ = 0.0072, β = 0.00125, and α = 0.000187, the dose-response curve would look like the blue line in Figure 1. The excess risk would have a “negative” area up to about 140 f/cc-years, meaning that the response would not be expected to be above baseline. The risk would then increase with exposure up to about 600 f/cc-years. The mathematical structure of Equation 1 assumes that, at very high exposure levels (not shown in Figure 1), the mesothelioma rate would start dropping because of the increased death rate in the population.

### Quantitative estimation of thresholds for mesothelioma using epidemiology data

#### Threshold simulation for mesothelioma cohort studies

Hodgson and Darnton (6) estimated average cumulative asbestos exposures in occupational cohorts. They then calculated excess mesothelioma mortality risk associated with exposures to chrysotile, amosite, and crocidolite. Darnton (21) updated this analysis using studies with increased follow-up in cohorts evaluated in 2000 and other cohorts for which data became available after the original analysis was conducted.

The goal of these analyses was to compare relative potency of these three fiber types, and not to identify whether a threshold exists. We used the data from Darnton (21) to test the plausibility of a threshold in the dose-response relationship for mesothelioma.

Mesothelioma mortality data for each of the non-textile chrysotile cohorts used by Darnton (21) were modeled by using a Poisson distribution and cumulative exposure levels were modeled as a uniform distribution with exposures ranging from 30% below the average value to 30% higher than the average value. An additional datapoint for a Russian cohort was also used. We generated linear models of the number of mesothelioma deaths per total expected deaths using following the equation:


(2) $$\begin{array}{l} M/TM\;\times 100\;=\;A+B\;*\;CE \end{array}$$

where M is number of mesothelioma cases in the cohort, TM is the total expected mortality from all causes, and CE is cumulative exposure.

A Monte Carlo simulation was used to determine various values of A and B for each combination of M and CE; TM was assumed to have a singular point value, as reported by Darnton (21). The linear regression model was considered to represent a threshold relationship if A ≤ 0 and B > 0. The level of the threshold for each model was defined as –A/B (f/cc-years).

**Figure 2:**
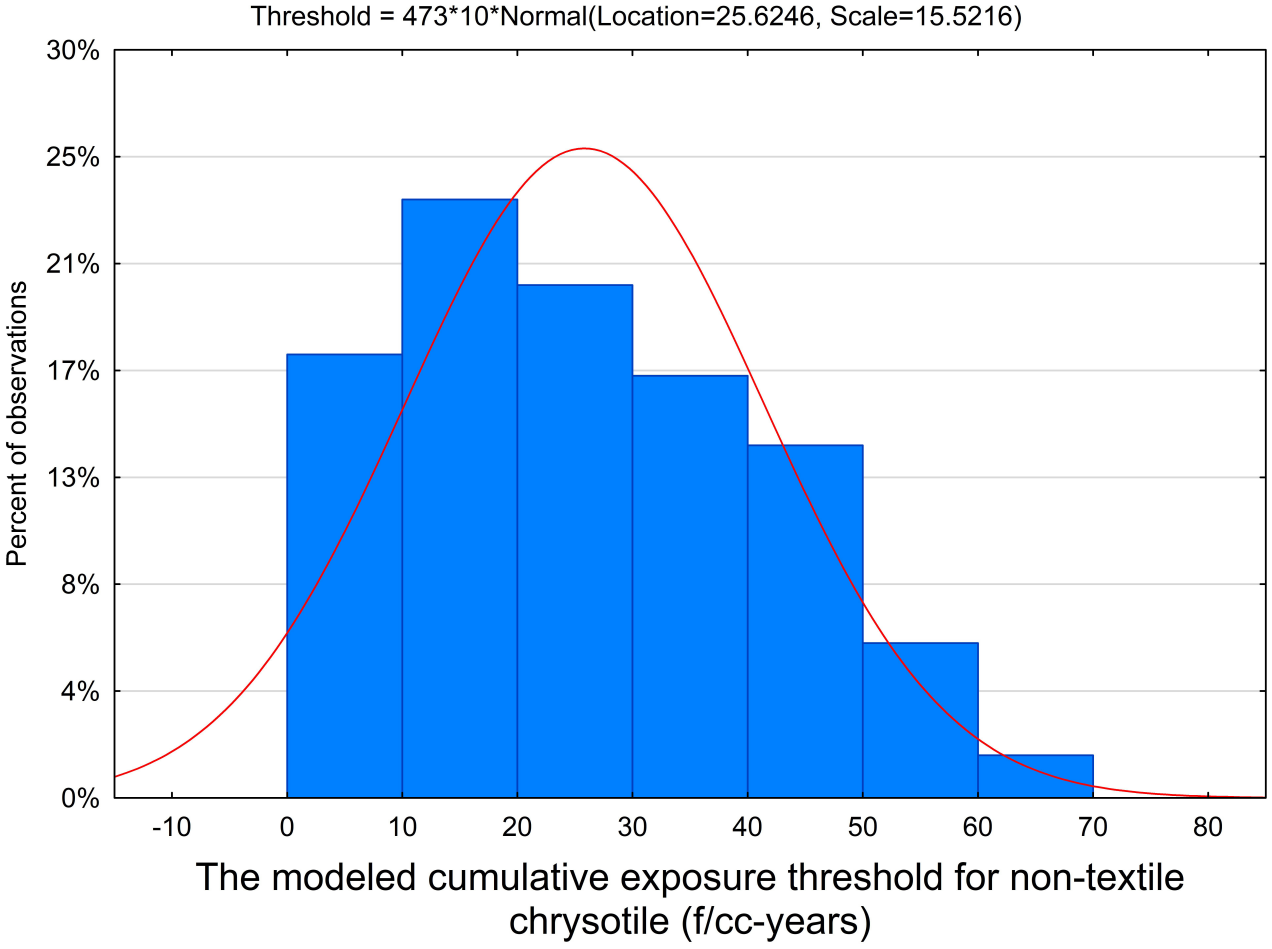
The distribution of threshold levels for non-textile chrysotile cohorts.

The fraction of threshold relationships among all generated models was determined, and the average threshold was estimated for all threshold relationships. We then calculated, from all combinations of parameters, 72% of models support thresholds. Also, the average threshold value of 25.6 f/cc-years was found (95% confidence interval [CI]: 24.2–27.1), with 5th and 95th percentiles of 3.3 and 52.9 f/cc-years, respectively. The average slope factor of the model was 0.0016 (95% CI: 0.0015-0.0017). This is depicted in Figure 2.

#### Threshold dose-response model for intensity and duration of exposure

Korchevskiy and Korchevskiy (25) demonstrated that, based on Peto et al. (60) model of age-related mesothelioma mortality, the lifetime risk of mesothelioma can be calculated by the formula:

Mesothelioma risk = R (V, Y, D, E) =


(3) $$\begin{array}{l} \int_{0}^{V-Y}I_{M}(t,\;d,\;E)dt=\int_{10}^{10+d}K_{M}E\;{\left(t-10\right)}^{3}dt\;\\ \;+\int_{10+D}^{V-Y}K_{M}E({\left(t-10\right)}^{3}-{\left(t-10-D\right)}^{3})\;dt=\\ \int_{10}^{V-Y}K_{M}E{\left(t-10\right)}^{3}\;dt-\int_{10+D}^{V-Y}K_{M}E{\left(t-10-D\right)}^{3}dt=\\ \frac{1}{14}xK_{M}\;x\;E\;x\;[{\left(V-Y-10\right)}^{4}-{\left(V-Y-10-D\right)}^{4}] \end{array}$$

where V is lifespan (years), Y is the exposure onset age (years), D is the exposure duration (years), E is the average exposure intensity (f/cc), and K_M_ is the coefficient (potency factor). A lag of 10 years was assumed to be consistent with the lag used in the original model by Peto et al. (60).

The mesothelioma risk model with threshold can be fitted to data from Table 1 utilizing the following expanded equation:


(4) $$\begin{array}{l} R(V,Y,D,E)=\frac{1}{14}xK_{M}\;x\;(E^{1.5}-Th)\;x\;[{\left(V-Y-10\right)}^{4}\\ \;-{\left(V-Y-10-D\right)}^{4}] \end{array}$$

with Th denoting the threshold by exposure intensity to the 1.5^th^ power, corresponding to a threshold value (the risk increment would be below zero if *E*^1.5^−*Th* < 0). We fit the data from Table 1 to Equation 4. The following parameters were found to produce the best fit:

K_M_ = 0.0042 × 10^−8^

V = 85.9 years

Th = 2.8 (R = 0.993, R^2^ = 0.98, *p* < 0.005)

The estimated risk value is 0 when Th = 2.8 and the exposure intensity is 2.8^1/1.5^ ~ 2 f/cc.

**Figure 3:**
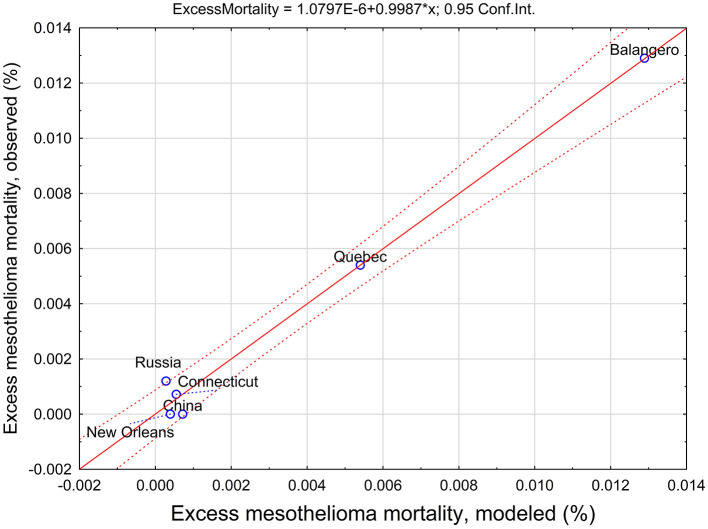
Observed and predicted excess mesothelioma mortality estimated using a threshold-based exposure intensity and duration model.

The relationship between observed and modeled data for mesothelioma mortality in chrysotile cohorts is illustrated in Figure 3.

An exposure intensity threshold of 2 f/cc corresponds to a cumulative exposure threshold of 90 f/cc-years, assuming maximum exposure duration of 45 years.

A longer minimal latency can also be tested to fit the data. For example, for a 20 year lag, the following equation can be used:


(5) $$\begin{array}{l} R(V,Y,D,E)=\frac{1}{14}xK_{M}\;x\;(E^{18}-Th)\;x\;[{\left(V-Y-20\right)}^{4}\\ \;-{\left(V-Y-20-D\right)}^{4}] \end{array}$$

with

K_M_ = 0.1 × 10^−8^

V = 99.9 years

Th = 2.0 (R = 0.994, R^2^ = 0.98, *p* < 0.006)

In this case, the estimated risk value is 0 when Th = 2 and the exposure intensity is 2.0^1/1.8^ ~ 1.5 f/cc. However, the fit of the model with a longer minimal latency is slightly worse than for the fit of Equation 4.

#### The “filter model” for the mesothelioma dose-response relationship

The “filter model” for cancer risk was developed in 1980 by Shaeffer et al. (61) at the United States Environmental Protection Agency (US EPA). We applied the model to mesothelioma mortality data by Darnton (21) for non-textile exposures, with the addition of the new datapoint for Russian mesothelioma study by IARC (24). The filter model is based on the Lagrangian Poisson Process for chromosomal aberrations from radiation exposure and, according to Schaeffer et al., it can be applied to all carcinogens. The model implies the presence of a threshold that can be found from its parameters. Schaeffer et al. (61) demonstrated that the model provided a good fit for various data on ionizing radiation, and also on ethylene oxide and vinyl chloride.

When applied to mesothelioma, the response (R) is modeled as a function of dose (D):


(6) $$\begin{array}{l} S\;=\;c\;+\;aln\left(CE+1\right)\;+b{\left[\ln\left(CE+1\right)\right]}^{2} \end{array}$$

where S is the lifetime fraction of cohort member without mesothelioma (“survival rate function”), CE is the cumulative exposure, and a, b, and c are coefficients.

Table 1 contains the data for the non-textile chrysotile cohorts, including cumulative exposures (f/cc-years), total expected mortality, numbers of mesothelioma cases, and the S values (“survival rate function”).

According to Schaeffer et al. (61), the threshold for the relationship between exposure (CE) and the survival function (S) would be achieved when


(7) $$\begin{array}{l} CE=exp\left(1-a/\left(2b\right)\right) \end{array}$$

when the second derivative of S by CE is equal to zero and changes its sign.

We fitted Equation 7 to the data for non-textile chrysotile (Table 1). The following parameters were determined:

a = 0.014

b = -0.00168

c = 0.972 (R = 0.91, R^2^ = 0.83, F = 19.7, *p* < 0.011).

**Figure 4:**
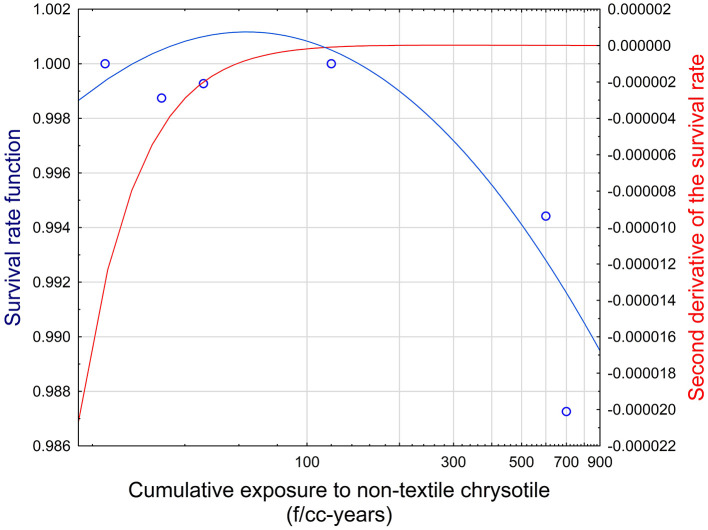
Survival function and its second derivative in relationship to cumulative exposure to non-textile chrysotile asbestos. Blue circles—Datapoints for cumulative exposure and mesothelioma mortality. Blue line—Survival rate function (left Y axis). Red line—Second derivative of the survival rate (right Y axis).

This combination of parameters corresponds to a threshold value for chrysotile of 162 f/cc-years. The establishment of a threshold is demonstrated in Figure 4. It can be seen that the fluctuation of the survival rate at the level below approximately 100 f/cc-years is not statistically consistent (i.e., no trend is observed). It is reflected in the negative second derivative of the survival rate function. The inflection point can be found at the level of cumulative exposure at about 162 f/cc-years, when the second derivative is equal to zero. Starting with this point, the trend in the decrease of the survival rate can be established. It corresponds to Schaeffer's concept, suggesting that the inflection point in the survival curve is an indication of a threshold. At the inflection point, the direction of the relationship between dose and response changes.

#### Determination of a possible mesothelioma threshold for other mineral fiber types

The quantification of an exposure threshold for amphiboles is complex because of the limited number of datapoints available for analysis. For example, three datapoints are available for crocidolite, three for amosite, but only one for Libby amphibole asbestos (LAA), the latter of which is often used as a surrogate for tremolite.

For non-textile chrysotile, several threshold estimates were derived above based on various models. In particular, a threshold simulation yielded a value of 25.6 f/cc-years (with an upper bound of about 53 f/cc-years). The exposure intensity and duration method yielded an upper-bound threshold of 90 f/cc-years. The filter model resulted in a threshold of 162 f/cc-years. The average level of about 90 f/cc-years can serve as a possible estimate of a threshold for non-textile chrysotile exposure.

Various approaches can be used to recalculate the proposed threshold levels for other mineral fiber types. For example, Beckett et al. (23) suggested that no observed adverse effect levels (NOAELs) for various mineral types of asbestos can be seen as proportional to mesothelioma slope (potency) factors as reported by Darnton (21). In this case, the threshold for crocidolite would be lower than for chrysotile by factor of 364, for amosite by factor of 86, and for LAA by factor of 21. Based on these proportions, the threshold for LAA is 4.3 f/cc-years, 1.04 f/cc-years for amosite, and 0.25 f/cc-years for crocidolite.

Thresholds can also be estimated for other non-regulated asbestiform minerals. For example, fluoro-edenite is known to produce an elevated risk of mesothelioma (62). Korchevskiy et al. (74) used epidemiology data and modeling to estimate fluoro-edenite mesothelioma potency as R_M_ = 0.12%. This estimate would lead to a threshold of 1.08 f/cc-years for fluoro-edenite. However, a comprehensive epidemiology study of populations exposed to fluoro-edenite would be needed to further substantiate this threshold.

We note that, while our estimate of thresholds for various mineral types in this case depends on the ratio of potency between amphibole and chrysotile fibers, this ratio was confirmed in various studies. For example, the ratio between crocidolite and chrysotile potency were consistent between Hodgson and Darnton (6) and Berman and Crump (50, 63), as well as with the most recent publications by Darnton (21) and Korchevskiy et al. (74).

The following logic can also be used to estimate the threshold for LAA, based on the filter model. We only had one datapoint for LAA. We assumed that 85 f/cc-years of exposure corresponds to 15 cases of mesothelioma per 574 total expected deaths and 0.974 survival function S. For coefficients a, b, and c, it would mean that:


(8) $$\begin{array}{l} 0.97\;\approx \;c\;+\;aln\left(16\right)+bln^{2}\left(16\right)\;=c\;+2.77a+7.67b \end{array}$$

If c fluctuated from 0.9 to 1 [according to Schaeffer et al. (61), who proposed that c should reflect a baseline survival function], and a from−0.87 to 1.3 (the range of a values in Schaeffer's paper), we can determine the threshold for LAA at the average level of 10.88 f/cc-years, with a standard deviation of 2.8.

### Mesothelioma threshold based on lung burden studies

Asbestos is ubiquitous in the environment and most, if not all, people have some level of exposure. The Agency for Toxic Substances and Disease Registry (ATSDR) (28) reported that ambient outdoor air concentrations for asbestos ranged from 0.000003 to 0.0003 f/cc and that ambient levels can reach 0.003 f/cc near local sources of asbestos (e.g., naturally occurring asbestos formations or facilities that mine, mill, or manufacture asbestos-containing products). Some populations residing near these local sources may have environmental or neighborhood exposures to asbestos that can result in higher risks for asbestos-related diseases.

Abelmann et al. (4) aggregated data from 17 published and unpublished studies and datasets that included 2,058 samples collected from urban, rural, or unknown locations from throughout the US. The authors adjusted for different analytical techniques and included only fibers ≥5 μm in length in their analyses. They estimated an overall mean from all sample locations from the 1960s to the 2000s of 0.00093 f/cc and a median of 0.00022 f/cc. ATSDR (28) estimated, based on ranges of typical indoor and outdoor exposures in both rural and urban areas, that cumulative exposure to asbestos over a lifetime (70 years) for the general population was 0.002-0.4 f/cc-year.

Knowing that all people have some background exposure to asbestos, we sought to determine whether there could be a threshold in lung tissue burden for mesothelioma. We found two papers in particular that provided information to address this.

Gilham et al. (64) evaluated asbestos fibers >5 μm in length in the lungs of mesothelioma and lung cancer patients. While it is possible that some lung cancers were caused by asbestos, the authors assumed that most were not. Based on transmission electron microscopy analyses, 57.7% of lung cancers had 0 to <0.025 million fibers ≥5 μm in length per dry gram of lung (average 0.00918 million fibers per dry gram).

Similarly, Rödelsperger et al. (65) collected lung tissue samples and measured asbestos exposures per dry gram of lung. For fibers >5 μm in length per dry gram of lung tissue, the authors divided study participants into five categories of asbestos lung burden: <0.05, 0.05 to <0.1, 0.1 to <0.2, 0.2 to <0.5, or ≥0.5 million fibers. The authors did not report a statistically significant increased risk of mesothelioma in the 0.05 to <0.1 million amphibole fibers >5 μm in length per dry gram of lung tissue vs. the lowest exposure category (odds ratio [OR] = 2.4, 95% CI: 0.8–7.6), but this association was statistically significant in the next higher exposure category (OR = 4.5, 95% CI: 1.1–17.9). These are very wide CIs, indicating that these estimates are statistically unstable. However, they still demonstrate a likely threshold.

## Discussion

The scientific arguments in support of thresholds for carcinogenic substances has gained support in recent decades, and the idea that any exposure to specific agents, no matter how small, can cause an elevated cancer risk appears to be exceedingly conservative. Several authors have argued that the linear non-threshold model for cancer, widely accepted in many countries for regulatory purposes, is not supported by biological observations (66–68).

Mesothelioma is a rare aggressive cancer that requires a unique combination of random factors for its initiation and development. It is reasonable to conclude that mesothelioma risk is not elevated when thresholds for certain factors are not exceeded, with some factors being quantitative (e.g., cumulative exposure level) and some qualitative (e.g., the presence or absence of amphibole fibers associated with chrysotile).

As noted above, Abelmann et al. (4) estimated that the lifetime ambient (general US population) cumulative exposure to asbestos ranged from approximately 0.002 to 0.4 f/cc-years over a 70-year lifetime. This exposure is mostly to chrysotile, with some possible fraction of amphibole asbestos. This range of values demonstrates that there should be a measurable level of asbestos exposure without an associated elevated baseline rate of spontaneous mesothelioma.

For various mineral fiber types, the minimum level of exposure associated with elevated mesothelioma risks is likely higher. In 16 studies analyzed by Beckett et al. (23), the NOAEL for mesothelioma from chrysotile with <10% of amphibole contamination was reported in the range from 100 to <400 f/cc-years to 800-1599 f/cc-years. While there is a discrepancy between the ranges reported by Beckett et al. (23) for an apparent threshold and the average levels of chrysotile exposure in cohort studies [reported by Darnton (21) or Berman and Crump (63)], it is useful to see that existing data support quite a high level of chrysotile exposure that is not associated increased mesothelioma rates.

We report several characteristics of silicate mineral fibers from the literature that can be used to define a qualitative threshold for mesothelioma mortality. These include (1) a minimum fiber width of 60 nm; (2) a maximum width between 1,000 and 100 nm; (3) lengths longer than 5 μm; (4) a proportion of the EMP fiber exposure made up of EMPA fibers longer than 5 μm with width ≤ 0.15 μm; (5) DCC ≥ 0.05, with the mixed category comprising fibers with DCC ≥ 0.01; and (6) a rigidity index greater than a threshold of 0.05 μm^2^ × GPa × 10^4^.

Several characteristics of mesotheliomagenic fibers allows researchers to distinguish them from non-mesotheliomagenic particles, which resemble nuisance dust rather than commercial asbestos (e.g., cleavage fragments). For example, the fraction of EMPA in cleavage fragments is typically zero. DCC for all analyzed cleavage fragments is below 0.05 (53). Another example is fibrous talc with a low Young's modulus, making its rigidity index much lower than a threshold of 0.05 μm^2^ × GPa × 10^4^ for elevated mesothelioma risk (39).

After analyzing the qualitative characteristics of a threshold, we developed a conceptual, theoretical model of a threshold for mesothelioma. We showed that a threshold can be observed as the probability of mesothelioma based on: (a) the probability of inflammation, (b) the probability of cell survival, and (c) the probability of the secretion of immunosuppressive and/or cell immortalization proteins. Each factor depends on the exposure level, but the probabilistic outcome would not exceed baseline rate of mesothelioma before the exposure reaches a specific level (threshold).

We also tested several statistical models using epidemiology information from non-textile chrysotile cohorts, including cohorts previously analyzed by Darnton (21), as well as the additional datapoint for the IARC study on chrysotile workers in Russia (24). We introduced error terms on the exposure levels and modeled number of and observed mesothelioma cases as random distributions. Linear models were fitted to the data in Monte Carlo simulations, reflecting various combinations of variables with consideration of errors. It was demonstrated that, from all simulations, 72% of all fitted models supported threshold vs. 28% that supported non-threshold models. The average threshold value of 25.6 f/cc-years was determined (95% CI: 24.2–27.1), with 5th and 95th percentiles of 3.3 and 52.9 f/cc-years, respectively. The average slope factor of the model was 0.0016% (95% CI: 0.0015–0.0017). It should be noted that Darnton (21) reported a slightly lower, though comparable, slope factor for all types of chrysotile (0.0014%), with an even lower slope factor for non-textile chrysotile (0.0011%). This was an expected outcome, because the threshold term was expected to elevate the slope factor of non-textile chrysotile for the levels exceeding the threshold.

We also used a non-linear model for approximating the lifetime mesothelioma risk by intensity and duration of exposure, based the model developed by Peto et al. (60). We demonstrated that the best fit corresponds to s model with a threshold for chrysotile exposure intensity to the 1.5th power, with the threshold level at 2 f/cc. This level corresponds to about 90 f/cc-years of lifetime exposure to non-textile chrysotile.

We also utilized the “filter” model, developed by Schaeffer et al. (61) from US EPA for all types of carcinogenic substances. The model implies that inflection points exist for carcinogenic dose-response relationships, reflecting a change from datapoints where excess risk is zero to a slope with a definite increasing relationship with higher levels of exposure. In our study, we applied the model to mesothelioma data and concluded that the approach by Schaeffer et al. (61) yields an estimated threshold for non-textile chrysotile at the level of 162 f/cc-years.

When several dose-response models are applied to specific epidemiology data, their combination requires specific consideration to determine a possible threshold. If we assume that various dose-response models can be averaged to determine the best estimates for lower exposure ranges [as demonstrated in several studies reviewed by Korchevskiy (69)], the best threshold estimates can be averaged to get the most reliable estimate. We suggest from this standpoint that a value of 90 f/cc-years can serve as a central tendency of a cumulative exposure threshold for chrysotile. From here, different approaches can be used to determine a threshold for other mineral types of fibers. We suggest threshold levels for amosite of 1.04 f/cc-years and for crocidolite of 0.25 f/cc-years. We also developed two different estimates of thresholds for LAA: 4.3 f/cc-years (standard deviation unknown) and 10.88 f/cc-years (standard deviation of 2.8).

The threshold estimates in our study can be compared to other published sources. The threshold of 90 f/cc-years for chrysotile is lower than the estimates reported by Beckett et al. (23); however, it is between the lowest average value for cohort studies [46 f/cc-years in the Darnton (21) meta-analysis] and NOAEL values reported by Pierce et al. (70) (208–415 f/cc-year). Recently, Willis et al. (71) published an assessment of cumulative exposure in workers with pleural and peritoneal mesothelioma from an amosite asbestos factory in Tyler, Texas. The minimal exposure value was found to be ~1.96 f/cc-years for pleural cases and 14.1 f/cc-years for peritoneal cases. The level for pleural cases is very similar to what we calculated for amosite (1.04 f/cc-years).

We also explored the existence of a threshold for asbestos lung burden in humans. This threshold may be seen as a function of a possible exposure threshold. Based on the Gilham et al. (64) and Rödelsperger et al. (65) studies, the level of 0.1 million amphibole fibers >5 um in length per dry gram of lung tissue can be seen as a possible threshold for mesothelioma.

The biggest strength of our study is that we estimated possible mesothelioma thresholds from several different angles. The consistency of the statistical threshold for mesothelioma can be observed from standpoints of mineralogical analysis, biological and theoretical modeling, estimation by various dose-response epidemiology-based models, and from lung burden studies.

That being said, the difference between empirically derived and model-based thresholds for mesothelioma should be interpreted correctly. All of our threshold estimates are model-based, though the models are derived from empirical data. In this capacity, the proposed thresholds should be used in conjunction with specific risk assessment models. For example, the suggested thresholds for mesothelioma can be used in the Hodgson and Darnton (6) risk calculation method. For example, a model-based excess risk estimate of mesothelioma for non-textile chrysotile can be used in the following equation:


(9) $$\begin{array}{l} Excess\;Risk\;\left(per\;10,000\;workers\right)\;=\;F\left(RM^{*}x\;\left(CE-Th\right)\;x\;10,000\right) \end{array}$$

where

Excess Risk = excess mesothelioma mortality

CE = cumulative exposure (f/cc-years)

Th = model-based threshold (f/cc-years)

RM^*^ = corrected slope factor for non-textile chrysotile

F(x) = function of argument x that is equal to x when x≥0, or 0 if x <0

In our study, we have not provided estimates for a corrected slope in the Hodgson and Darnton (6) method, focusing instead on estimating thresholds. Corrected slopes will be proposed in the follow-up studies.

Our study has uncertainties and limitations. There are various arguments that the existence of a threshold cannot be scientifically postulated only based on data (72). However, if a threshold-based dose-response model is established, the determination of a threshold is certainly possible. Moreover, as we noted above, the benchmark dose approach claims to be a tool of choice to reconstruct risk level at low exposures based on mathematical fitting for high-exposure datapoints. Another limitation of our study is that the models were tested on non-textile chrysotile cohorts only. It would be beneficial to expand the study to other available information for different mineral types, but the available data for other mineral types are even more limited.

In addition, the estimates of amphibole thresholds in our study were based on the ratio of chrysotile to amphibole potency factors, as reported by various authors. In particular, asbestiform crocidolite is one of the most potent fibers for mesothelioma. Darnton (21) reported crocidolite's potency for mesothelioma as 0.52% (95% CI: 0.47–0.58), and the potency for non-textile chrysotile as 0.0011% (95% CI: 0.00079–0.0014). This difference between the potency factors drives our estimates of thresholds, and the mesothelioma threshold for crocidolite we estimated is significantly higher than that for chrysotile. The limitation of the approach, as it was emphasized above, is in the utilization of a slope factor as a parameter, reciprocal to the threshold value. We expect that this aspect of our analysis will be further expanded in the future.

We assume that further developments can be expected for the study of thresholds in mesothelioma. Biological mechanisms of mesothelioma development can be explored more deeply, and *in vitro* data can be used to establish a lowest level of exposure for several important biological reactions (e.g., producing elevated HMGB-1 levels in tissues exposed to asbestos fibers). Additional sets of models can be applied to epidemiology data to determine a threshold. In particular, the Hill model can be tested to reflect multi-phase features of mesothelial carcinogenic process (73). The role of uncertainty and errors in exposure and response assessment can be further studies for the purpose of statistical evaluations of thresholds. Lung burden studies also are expected to contribute additional values for threshold determination. In particular, better relationships between cumulative exposure and lung burden can help in the evaluation of a threshold based on the data from pathology evaluation.

In conclusion, we have demonstrated that, while uncertainties remain, many angles of scientific evidence support the existence of a threshold for mesothelioma. Our preliminary estimate of a central-tendency cumulative exposure threshold level for non-textile chrysotile is ~90 f/cc-years. Based on our proposed approach, we suggest thresholds of 1.04 f/cc-years for amosite, 0.25 f/cc-years for crocidolite, and 4.3–10.9 f/cc-years for tremolite. Future studies should be conducted to support these estimates.

## Data Availability

The original contributions presented in the study are included in the article/supplementary material, further inquiries can be directed to the corresponding author.

## Footnotes

^1^Young's modulus describes the relationship between stress and strain (*i.e*., how easily a material can bend or stretch).
